# A Missing Link: Engagements of Dendritic Cells in the Pathogenesis of SARS-CoV-2 Infections

**DOI:** 10.3390/ijms22031118

**Published:** 2021-01-23

**Authors:** Abdulaziz Alamri, Derek Fisk, Deepak Upreti, Sam K. P. Kung

**Affiliations:** 1 Department of Immunology, Rady Faculty of Health Sciences, Max Rady College of Medicine, University of Manitoba, Winnipeg, MB R3E OT5, Canada; alamria3@myumanitoba.ca (A.A.); fiskd@myumanitoba.ca (D.F.); 2 Surgery, Faculty of Health Sciences, McMaster University, 1200 Main Street West, Hamilton, ON L8N 3Z5, Canada; rajdpak@gmail.com

**Keywords:** COVID-19, SARS-CoV2, dendritic cells, immunopathology, therapy

## Abstract

Dendritic cells (DC) connect the innate and adaptive arms of the immune system and carry out numerous roles that are significant in the context of viral disease. Their functions include the control of inflammatory responses, the promotion of tolerance, cross-presentation, immune cell recruitment and the production of antiviral cytokines. Based primarily on the available literature that characterizes the behaviour of many DC subsets during Severe acute respiratory syndrome (SARS) and coronavirus disease 2019 (COVID-19), we speculated possible mechanisms through which DC could contribute to COVID-19 immune responses, such as dissemination of Severe acute respiratory syndrome coronavirus 2 (SARS-CoV-2) to lymph nodes, mounting dysfunctional inteferon responses and T cell immunity in patients. We highlighted gaps of knowledge in our understanding of DC in COVID-19 pathogenesis and discussed current pre-clinical development of therapies for COVID-19.

## 1. Introduction

Since the outbreak of coronavirus disease 2019 (COVID-19 ) in December 2019, over 70 million cases of COVID-19 have been reported worldwide, with more than 1,500,000 lives taken by the disease (https://www.ecdc.europa.eu/en/geographical-distribution-2019-ncov-cases). Severe acute respiratory syndrome coronavirus 2 (SARS-CoV-2) was identified to be the virus that caused the disease. SARS-CoV-2 shares 79% and 50% nucleotide homology with SARS-CoV-1 and MERS-CoV, respectively [1]. A total of 80% of those who had acquired COVID-19 had mild infections, characterized by nonspecific and minor symptoms. Also, 15% of cases were moderate, with pneumonia, hypoxia, pulmonary inflammation and the need for hospitalization. The remaining 5% experienced severe disease, composed of acute respiratory distress syndrome (ARDS) and systemic hyperinflammation. Finally, 1–2% of the confirmed COVID-19 cases have experienced fatal outcomes [2]. In severe disease, increased levels of various cytokines and chemokines constitute a “cytokine storm,” in which systemic inflammation causes damage to multiple organs of the body [3]. Dysregulated production of these cytokines and chemokines can drive the recruitment of inflammatory neutrophils and monocytes into lung tissue, contributing to the development of ARDS [4]. In addition, lymphopenias in CD8+ T cells and CD4+ T cells are common and correlate with disease severity [5,6,7,8]. Of interest, the roles and functions of dendritic cells (DC) during SARS-CoV-2 infection have not yet been fully understood.

DC are professional antigen-presenting cells (APCs) that have the ability to mediate innate immunity and shape adaptive immunity. Upon stimulation, DC undergo a maturation process that enables their migrations to secondary lymphoid organs where they activate and shape T cell differentiations [9]. As a result, DC play pivotal roles in ensuring the induction of CD4+ and CD8+ T cell responses against various pathogens. A variety of DC subsets have been identified and their expression profiles have revealed remarkable heterogeneity. Among all of the DC subsets reported to date, plasmacytoid DC, conventional DC, monocyte-derived DC and Langerhans cells are the major DC subsets studied in most infection models (Table 1).

Many key findings were made concerning both DC function and immunopathology in patients with SARS. Emerging work suggested there could be several significant ways in which DC may impact the outcomes of COVID-19 patients. In this review, we highlight the potential role(s) of DC in SARS-CoV-2 dissemination, as well as innate and adaptive immune responses to the virus. We also discuss deviations in DC subset numbers that have been observed in patients with the disease. Lastly, we incorporate the findings highlighted in this review into an evaluation of therapies being currently investigated and pose potential DC-related therapeutic options that could add to the arsenal of treatments being considered for COVID-19.

## 2. A Possible Role of DC in SARS-CoV-2 Virus Dissemination

In addition to the severe pulmonary pathology observed in many COVID-19 patients, low copy numbers of SARS-CoV-2 RNA have been detected at autopsy in other organs, including the kidneys, testicles, heart and brain [18], although replication of the virus in these organs is yet to be confirmed. Nonetheless, the presence of viral RNA beyond the lungs may indicate that the virus can spread to other organs through the bloodstream [19]. The spread of virus to lymphoid organs could have significant effects on disease outcome. SARS-CoV-1 infected and destroyed T cells more quickly than HIV [20], making it important to confirm whether SARS-CoV-2 functions similarly. T cell lymphopenias in particular have been correlated to COVID-19 severity [8]. SARS-CoV-2 may promote such T cell lymphopenia directly [21] or indirectly upon its dissemination at the distal sites. It is established that dissemination of HIV to the lymph nodes is facilitated by DC. After HIV infects DC, they migrate to dLNs to trans-infect CD4+ T cells [22]. Similar to HIV-1, SARS-CoV-1 and SARS-CoV-2 S proteins have been found to bind DC-SIGN [23,24,25]. In vitro, DC infected with HIV gp160-pseudotyped lentivirus were shown to form infectious synapses with T cells [26]. Yang et al. attempted to infect THP-1 cells with a similar vector, psuedotyped instead with SARS-CoV-1 S protein. Only THP-1 that were made to express DC-SIGN were capable of both binding to and transferring the vector to Vero cells. When they proceeded to do the same with mature cDC2, confocal microscopy revealed the transfer of virus into 786-O (human renal) cells through infectious synapses. This process was found to be inhibited by anti-S protein mouse antiserum [24]. Their results suggest that S protein enabled cell-mediated transfer of SARS-CoV-1 into permissive cells by cDC2. Specifically, DC-SIGN might be one of many lectins that could contribute to this transfer. Recently, DC-SIGN was found to be up-regulated on moDC after being infected by SARS-CoV-2 [27]. Thus, if DC-SIGN is used by SARS-CoV-2 to facilitate its spread through DC migration, SARS-CoV-2 may up-regulate DC-SIGN to increase the efficiency of its dissemination. DCs might therefore spread SARS-CoV-2 to other cell types through cell-to-cell contacts in vivo.

Later, this idea was broadly explored in vivo using a macaque model. One observation made was that macaque Langerin+ DC (e.g., LC) express angiotensin-converting enzyme 2 (ACE2) more strongly than type II pneumocytes. LC were found to be infected by SARS-CoV-1. However, as was seen for HIV, the infection was also non-productive. Infected LC most noticeably migrated to the tonsils, where a productive infection became established. SARS-CoV-1-infected cells also migrated to the lymph nodes 2 days post-in fection (d.p.i.) and systemic dissemination occurred afterwards (7 d.p.i.). These observations were followed up by co-culturing SARS-CoV-1-infected cDC with Vero E6 cells, a cell line that is permissive to SARS-CoV-1 infection. Viral foci formed near the membranes of infected cDC, where they were transferred into Vero E6 cells in a S protein-dependent manner [28]. This observation demonstrated that infected DC might spread these coronaviruses to permissive cells throughout the body during and/or after migration to LNs, where the virus may gain access to the bloodstream for travel to various organs.

Key questions for SARS-CoV-2 are pending: Is DC migration activated or inhibited in vivo upon encounter with SARS-CoV-2? Do the infected DC trans-infect permissible cell types? Which DC subsets aside from moDC (i.e., pDC, cDC, LC,) can be infected by the virus? If any, are these infections productive?

## 3. DC and Innate Immunity to COVID-19

In severe COVID-19, innate immunity has been characterized as an excessive, pro-inflammatory response to the virus. Significantly elevated amounts of various cytokines and chemokines constitute a “cytokine storm,” which has been directly correlated to multi-organ failure, lung injury and poorer prognosis [29]. Cytokines and chemokines found to be elevated in COVID-19 patients include (IL)-2, IL-2Ra, IL-6, IL-7, IL-8, IL-10, IL-12 IL-17, tumor necrosis factor alpha (TNF-α), monocyte chemoattractant protein-1 (MCP-1), macrophage inflammatory protein-1 alpha (MIP-1-α), granulocyte colony-stimulating factor (G-CSF) and C-X-C motif chemokine ligand 10 (CXCL10), also known as IFN-γ inducible protein (IP-10) [3,4,30]. In addition, inflammatory cells infiltrate the lungs, where they can cause extensive tissue damage through the release of reactive oxygen species and proteases [31].

Given the abilities of DC to control/regulate inflammatory responses [32], dysregulated DC may contribute to the harmful innate immune responses that occur in patients with severer disease. While cDC and pDC express little to no ACE2 [33], moDC have recently been shown to express ACE2, albeit less than Calu3 cells [27]. SARS-CoV-2 has been demonstrated to infect moDC, but the infection is abortive [27,34]. In addition to ACE2, SARS-CoV-2 may infect various DC subsets through interaction with CD147, another receptor which binds SARS-CoV-2. The expression of CD147 by a non-susceptible cell line is sufficient to render it susceptible to infection [35] and it is expressed by DC [36]. moDC infected with SARS-CoV-1 produced significant amounts of pro-inflammatory chemokines, including MCP-1, MIP-1-α and IP-10. In addition, moderate increases in inflammatory cytokines TNF-α and IL-6 were observed, while expression of type I and II IFNs was low [37]. The differential expression of all of these cytokines and chemokines was measured during the in vitro SARS-CoV-2 infections of moDCs mentioned earlier. Interestingly, only IP-10 was significantly up-regulated [27]. Therefore, the functions of moDCs in response to SARS-CoV-2 might differ from moDC responses to SARS-CoV-1 by specializing more in the recruitment of inflammatory immune cells to the site of infection. In the context of COVID-19, many studies have observed a correlation between IP-10 and disease severity [38,39,40], and that IP-10 can serve as an excellent predictor of disease progression [38,41]. One study has indicated that IP-10 signaling through its chemotactic receptor, CXCR3, may be critical in the development of viral and non-viral ARDS [42]. Inflammatory neutrophils, Th1, natural killer (NK) cells, and eosinophils express CXCR3 [43,44] and could theoretically exacerbate local inflammation after recruitment to the site of infection by moDC-derived IP-10. On the other hand, a similar study noted that moDCs produce IFN-α, IFN-β, TNF-α, IL-6, IL-8, IL-10 and IL-1β after infection with live, but not UV-inactivated SARS-CoV-2. However, production of these cytokines was generally lower in comparison to SARS-CoV-2 monocyte-derived macrophage. Unlike earlier observations, this study observed minimal up-regulation of IP-10 [34]. Thus, in contrast to SARS-CoV-1, in vitro infections of DC show conflicting support for the idea that DCs act as a significant source of proinflammatory cytokines and point toward other cell types that may be contributors of the cytokine storms observed in severe COVID-19.

Decreases in pDC number have been observed in the peripheral blood and respiratory tract of COVID-19, but do not correlate with disease severity [45]. Still, pDC are likely integral to successful immune responses to SARS-CoV-2 due to their ability to produce massive amounts of type I IFN. One noteworthy observation is that SARS-CoV-2 is more sensitive to type I IFN in vitro than SARS-CoV-1 [46]. Although in vitro responses of pDC to SARS-CoV-2 have not been examined, pDC infected with SARS-CoV-1 or MERS-CoV are strongly induced to produce type I IFN [47,48]. Type I IFN production by DCs infected with SARS-CoV-2 has only been evaluated in moDC thus far, and whether they upregulate type I, II or III IFN production following infection is still unclear. One study has revealed that IFN antagonism in moDC may occur due to inhibition of signal transducer and activator of transcription 1 (STAT-1) phosphorylation by SARS-CoV-2. Interestingly, STAT-1 phosphorylation in infected moDC was significantly more inhibited by SARS-CoV-2 than SARS-CoV-1 [27]. SARS-CoV-1 Nsp1 is an IFN antagonist that has been noted to inhibit STAT-1 phosphorylation, block host translation and specifically induce host mRNA degradation [49], all of which could contribute to diminished type I IFN production. Other SARS-CoV-1 viral proteins that were capable of inhibiting IFN responses include Membrane (M) protein, Nucleocapsid (N) protein, Nsp3, Nsp16, ORF3b and ORF6 [49]. Examination of the SARS-CoV-2 homologs in the regulation of type I IFN expression will be of great interest.

Previously, pDC-derived type I IFN was shown to prevent dissemination from the LNs of another β-coronavirus known as mouse hepatitis virus (MHV) [47]. Hence, one might speculate that type I IFN deficiency in some patients could contribute to SARS-CoV-2 viremia and, consequently, accelerate disease. A single-cell transcriptomic analysis has paradoxically observed that, although the production of IFN-α by pDC is ablated, interferon-stimulated gene (ISG) expression is enhanced in PBMCs. The extent of impaired type I IFN production by pDC does not appear to be a determinant of disease severity [50]. Yet, Hadjadj et al. found that excessive inflammatory responses and persistent blood viral loads in severe and critical COVID-19 patients are associated with low type I IFN production and activity. Type I production was nearly absent in critical disease, high but transient in severe disease and remained high in mild disease [21]. Similarly, in biopsies of COVID-19-associated perniosis from patients with severe or critical disease, the expression of a type I IFN-inducible gene (myxovirus resistance protein A) was absent, while its expression was high in biopsies from patients with mild disease [51]. If pDC are equivalently impaired in patients with mild and severe disease, future work should seek to understand which cell types may give rise to milder disease by substituting pDC as the primary type I IFN producer.

The nucleocapsid (N) protein of SARS-CoV-1 was capable of antagonizing IFN responses by physically blocking recognition of SARS-CoV-1 RNA by TLR3 [52]. Recently, Mu et al. reported that SARS-CoV-2 N protein physically binds dsRNA in 293T cells [53]. This suggests that DC immune responses may be inhibited to some extent by interference with TLR3 activation by SARS-CoV-2 N protein. Of interest, TLR3 activity in lung-resident DC is the likely source of IFN-λ that was found in the bronchoalveolar lavage fluid (BALF) of COVID-19 patients [54]. Contribution of DC in IFN responses, relative to other cell types, remains an interesting question to examine.

TLR7 and TLR8 are phylogenetically and structurally related endosomal PRRs that can trigger distinct downstream effects after binding to ssRNA, making them key players in the recognition and response to ssRNA viruses like SARS-CoV-2 [55]. Based on observations made relating to SARS-CoV-1, hyperinflammatory responses to COVID-19 may also be owing to TLR7 and TLR8 activity. In a murine model, injection of a GU-rich region of the SARS-CoV-1 genome that is recognized by TLR7 and TLR8 resulted in significant increases in TNFα, IL-6 and IL-12 levels. In addition, infiltration of inflammatory cells into the lungs, pulmonary edema and alveolar hemorrhage were observed. Interestingly, more than half of the mice inoculated with the RNA alone died within 48 h [56]. These effects may be even more relevant to severe COVID-19, as it has been reported that SARS-CoV-2 has more ssRNA fragments in its genome that can be recognized by TLR7 and TLR8 compared to SARS-CoV-1 [57]. Regarding DC, TLR7 is most clearly expressed in pDC, while TLR8 is expressed in moDC. TLR7 stimulation in pDCs results in large quantities of IFN-α being produced, which could serve as an antiviral response to viral genome recognition. On the other hand, moDC TLR8 signaling leads to an increase in the secretion of C-C motif ligand 2 (CCL2), CCL3, CCL4, CCL5, MCP-1, TNFα, IL-8 and IL-12p40, [58] some of which are yet to be characterized in in vitro infections of moDC. It is tempting to speculate that the variety of chemokines produced by moDC in response to TLR8 stimulation could partially explain the infiltration of inflammatory cells into the lungs of mice treated with SARS-CoV-1 GU-rich ssRNA and even mice models of COVID-19. Of note, IFN-α production following TLR7 stimulation is greater in pDC from females than from males. It has been hypothesized that type I IFN production by pDC may control early SARS-CoV-2 infection in females better than in males, leading to better outcomes on average and explaining why 70% of those who die from the disease are male [59].

The proportions of three other DC subsets in patient sera appear to also be affected by COVID-19. Similar to pDC, cDC1 levels are diminished in peripheral blood samples with no correlation to disease severity and are undetectable via bronchoscopy [60]. If the numbers of cDC1 are depleted in the lungs and/or lymphoid tissues during SARS-CoV-2 infection, less cDC1 may be available for the activation of CD8+ T cell responses.

Much is still unknown about how DC respond to SARS-CoV-2. How do TLR members on specific DC subsets respond to SARS-CoV-2? Does N protein alone cause IFN antagonism in moDC and other subsets? More broadly, what are the cytokine/chemokine expression profiles of other DC subsets when challenged with SARS-CoV-2? Could other cellular processes, such as the apoptosis of DC, explain the decreases of some DC subsets in the blood of COVID-19 patients? Recently, some perplexing findings were obtained from a metatranscriptomic analysis of the upper airways of COVID-19 patients. While many genes related to immunity, including TLR cascades, IL signaling, inflammasome activity are up-regulated in response to many upper respiratory tract infections, this was not observed in COVID-19 patients [61]. These observations stir up a multitude of questions: is this pattern also representative of DCs situated in the upper respiratory tract? If so, does this differ from the expression profiles of DC situated in the lower respiratory tract? Do their results indicate immune evasion, aiding SARS-CoV-2 persistence in vivo? Or, on the contrary, does the decreased induction in these immunity pathways partly explain the lower mortality rate of COVID-19 compared to SARS?

## 4. DC and Adaptive Immunity toward COVID-19

In the adaptive immune response to SARS-CoV-2, virus-specific T and B lymphocytes play an essential role in controlling the infection [62]. The lymphopenia that has been observed in COVID-19 patients mostly reflects lower levels of CD4+ T cells and CD8+ T cells that correlate with increased disease severity [63]. The mechanisms behind T cell lymphopenias, as well as the reasons for T cell levels being more affected than B cells, are still incompletely understood [64].

Decreases in T cells in the periphery may simply indicate their migration out of the blood. However, if the absolute numbers of effector CD4+ T and CD8+ T cells in the body are reduced, impairment of viral clearance may follow, leading to the escalation of disease to critical stages. It was reported that chemokine receptors (CCR-1, CCR-3 and CCR-5) were significantly up-regulated by moDC infected with SARS-CoV-1 [65]. To date, differential regulation of DC chemokine receptors has yet to be examined through in vitro infections. So far, evidence of DC migration in COVID-19 patients is limited to the migration of cDC2 from the blood to the lungs. The decrease in cDC2 in the blood correlated with disease severity. In bronchoscopy samples, infiltration of CD38+ CD8+ T cells into the lungs correlated with the preferential recruitment of cDC2 relative to inflammatory transitional and non-classical monocytes. This finding indicated that the preferential infiltration of cDC2 out of the blood and into the lungs may assist cytotoxic T cell activity at the site of infection. Decreases in pDC and cDC1 were also observed, but these decreases were independent of disease severity and, if they were due to DC migration out of the blood, the destination of their migration was not determined [60].

In vivo, severe COVID-19 patients, including children who experience a Kawasaki-like, multi-system inflammatory syndrome in response to the virus have reduced expression of HLA-DR and CD86 in cDC [66,67]. Interestingly, cDC isolated from COVID-19 patients fail to up-regulate HLA-DR, CD86 and PD-L1 in response to stimulation with TLR agonists [67]. It is therefore possible that DC that migrate into the infected tissues are de-regulated in inducing anti-viral T cell responses. Thus far, characterization of DC from COVID-19 patients is reflective of suppressed antigen presentation. It has yet to be shown whether the heightened inflammatory environment in patients with severer disease specifically contributes to dysfunctional antigen presentation by DC and, consequently, reduced T cell stimulation. It was shown that the supernatant of Calu-3 cells, highly polarized human lung epithelial cells, infected with SARS-CoV-1 resulted in the up-regulation of CD40 and CD86, but not CD83 or MHC class II. Importantly, these moDC also completely lost their ability to stimulate naïve T cells [68]. It is therefore important to consider any indirect effects that SARS-CoV-2 infections have on the microenvironment that will in turn impact DC-mediated adaptive immunity.

A few other studies have showcased additional roles that DC may have in adaptive immune responses to SARS-CoV-2. Interestingly, tumor necrosis factor-related apoptosis-inducing ligand (TRAIL) is upregulated in SARS-CoV-1-infected immature moDC and TRAIL expression has correlated with SARS-CoV-2 viral loads in patients [65,69]. Therefore, infected DC might directly promote T cell lymphopenia by inducing apoptosis of T lymphocytes in LNs through TRAIL. A computational analysis predicted that DC produce IL-18, TNF superfamily member 13 (TNFSF13) and TNFSF13B to promote B and T cell proliferation in early COVID-19 recovery [70]. If confirmed in vivo, such findings would represent a function of DC that is operating efficaciously in COVID-19 patients.

**Figure 1:**
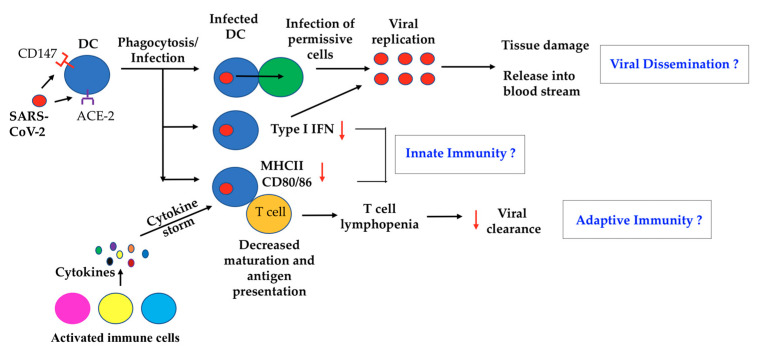
Potential dendritic cells (DC) Dysfunction(s) in coronavirus disease 2019 (COVID-19) patients. In viral dissemination, DC could be abortively infected by SARS-CoV-2. Infected DC may spread to infect permissive cells through trans-infection, or gain access to the bloodstream for dissemination throughout the body. In innate immunity, moDC infected with Severe acute respiratory syndrome coronavirus 2 (SARS-CoV-2) demonstrate antagonized type I IFN production. Reduced type I IFN levels facilitate viral replication at sites of infection. DC maturation has been found to be inhibited in vivo. In Adaptive immunity, compromised DC maturation could impair naïve T-cell primings and may indirectly contribute to observed T cell lymphopenias, which collectively can impair viral clearance. As diverse DC subsets have specific functional activities, DC subsets may also induce different functional responses to SARS-CoV-2 infection in vivo.

In conclusion, the roles of DC in COVID-19 could be further elucidated through the co-culturing of healthy DC with the supernatant of SARS-CoV-2-infected Calu-3 cells, or infected DC with healthy naïve T cells to determine if DC trans-infect, fail to activate or cause the apoptosis of T cells. In addition, measuring the up-regulation of co-stimulatory molecules, other maturation markers and chemokine receptors on immature cDC and moDC in response to SARS-CoV-2 infection would direct future in vivo investigations of DC functions during COVID-19 (Figure 1).

## 5. Integrating DC Biology into Potential Treatments for COVID-19

Currently, potential drugs targeting various aspect of SARS-CoV-2 infections have been examined in pre-clinical models and clinical cohorts. Although these approaches are not DC-specific, most of them could, in principle, impact DC as well (Table 2). It is therefore relevant to examine further how these treatments may impact DC biology that associate with disease outcomes.

Due to the reliance of SARS-CoV-2 on S protein-mediated membrane fusion to infect vulnerable cells, clinical trials are being conducted on the efficacy of anti-S protein monoclonal and polyclonal antibodies as treatments for COVID-19 (NCT04425629, NCT04454398, NCT04453384) and many developing vaccines are based on SARS-CoV-2 S protein (NCT04453852, NCT04368988). Because it was shown that anti-S protein not only neutralized SARS-CoV-1 but also prevented its trans-infection of vulnerable cells by DC [24], developing S protein-targeted treatments may also prevent any potential role of DC in trans-infecting permissive cell types. If such a role exists, it may be most effective to administer exogenous antibodies earlier during the duration of the disease to curb viral spread prior to disease escalation.

IL-6 blockade may also, in conjunction with other benefits, improve the T cell stimulatory capacity of DC. As mentioned earlier, IL-6 produced by Calu-3 cells infected with SARS-CoV-1 abolished the ability of moDC to stimulate naïve T cells [71]. Thus, IL-6 levels that tend to be highest in patients with worse outcomes may impair important T cell responses, lending more support to the use of IL-6 blockade in these patients. Two available drugs, sarilumab and tocilizumab, monoclonal antibodies that bind IL-6R and block IL-6 signaling, are currently available [72]. IL-6 receptor inhibitors are licensed for various autoimmune disorders and are considered easy to tolerate and safe to use. In fact, tocilizumab administration has been successful at immediately ameliorating clinical outcomes in patients with severe and critical disease [73]. A phase two clinical trial reported similar results, even with low doses of tocilizumab [74].

The use of corticosteroids in the treatment of COVID-19 has been debated. With regard to DC, corticosteroid administration appears to have negative consequences. SARS patients receiving higher doses of corticosteroids had lower peripheral levels of pDC (reduced by 28-fold), cDC, CD4+ T cells and CD8+ T cells [75]. On one hand, reduced levels in some of these cells could moderate disease severity by reducing the cellular sources of cytokine storms. On the other hand, early administration of corticosteroids may interfere with the production and timing of pDC-derived type I IFN, facilitating viral replication. Also of concern, the prolonged or premature use of corticosteroids might hinder adaptive immunity to and recovery from COVID-19 by reducing the levels of multiple subsets. It has been suggested that DC influenced by corticosteroids or IL-10 can induce tolerance via T cell deletion or induction of regulatory T cells [58,75]

GM-CSF is a cytokine with a cardinal role in the modulation of inflammation. Ligand binding to the GM-CSF receptor-α (GM-CSFRα) activates multiple pro-inflammatory pathways to increase the secretion of pro-inflammatory cytokines [76] and activates multiple downstream signaling pathways, which influence activation and differentiation of myeloid cells. Under physiological conditions, the concentration of circulating GM-CSF is low, but concentrations are elevated in inflammatory settings. Several cell types can serve as a source of GM-CSF, including DC, T cells, neutrophils, eosinophils and tumor cells, with most production occurring locally at the site of inflammation [76], thus functioning as a feed-forward inflammatory amplifier. It has been noted that GM-CSF is elevated in COVID-19 patients and is higher in patients with severe disease compared to those with mild disease [77]. The production of GM-CSF in patients has been described as disproportionate and delayed [78]. There is some evidence that GM-CSF triggers the differentiation of DC that ultimately have tolerogenic phenotypes and are capable of causing T cell hyporesponsiveness and/or anergy. Also, tolerogenic DC are capable of increasing Treg numbers and functions, which could increase tolerance to viral infection [79]. It has also been suggested that elevated GM-CSF levels may give rise to the distinct immunopathology observed in children with multi-system inflammatory syndrome due to SARS-CoV-2 infection. In Kawasaki Disease models, GM-CSF confers lung protection while also triggering cardiac pathology. Thus, GM-CSF activity might help explain why children with this manifestation of the disease develop cardiac pathologies (e.g., myocarditis), yet commonly lack respiratory findings [80].

Mavrilimumab is an anti-GM-CSFRα monoclonal antibody used to antagonize GM-CSF signaling. It was reported that administration of mavrilimumab in patients with COVID-19 pneumonia and hyperinflammation improved clinical outcomes compared with local standard care. The improvement of respiratory outcomes with mavrilimumab resulted in earlier weaning from supplemental oxygen and shorter hospital stays compared to standard care alone [81]. The reversal of inefficient DC phenotypes may be one of many possible cellular events that might explain the observed positive outcomes of mavrilimumab treatment.

Type I IFN is another treatment option that ought to be considered for COVID-19. The observation that pDC-ablated mice have increased viral dissemination of MHV to the liver, spleen and lungs demonstrates that pDC and the type I IFN they produce should be evaluated in the context of COVID-19, whose etiological agent belongs to the same group of coronaviruses [47]. A similar pattern may be present in COVID-19 patients; those with severer disease have been found to have reduced type I IFN in serum samples that is accompanied by persistent viral loads and inflammatory responses [21]. If an impaired pDC or type I IFN response contributes to sustained viral loads and exacerbated inflammation, the administration of type I IFN may be therapeutic. Indeed, IFN signaling may be even more important in the context of COVID-19 compared to SARS due to the higher in vitro sensitivity to type I IFN belonging to SARS-CoV-2 [46]. A clinical trial of IFN-α2β in COVID-19 patients demonstrated a reduced duration of elevated IL-6 and C-reactive protein (CRP) levels, as well as hastened viral clearance in the upper respiratory tract [82]. The administration of type I IFN may be effective for COVID-19 patients and its use seems justified at the level of DC.

The status and activity of some immune cells tend to be compromised in older individuals. For example, Scarfò et al. have found that patients with chronic lymphocytic leukemia (CLL) who contract COVID-19 face more severe symptoms if they are older. The majority of patients (73%) presented severe symptoms, and if the patients were aged 65 or older, they were more likely to have severe symptoms [83]. One trait associated with age is the diminished ability of DCs to produce type I and III IFN [84]. In addition to the administration of type I IFN, stimulating IFN production in vivo may be an alternative treatment avenue that is worth considering particularly for older patients. TLR3 agonism using polyinosinic:polycytidylic acid (poly I:C) up-regulates IFN-β and IFN-λ in some DC subsets. The prophylactic, intranasal administration of poly I:C in aged mice later infected with a mouse-adapted strain of SARS-CoV-1 resulted in complete protection (100% survival). It should be noted that this benefit was incredibly sensitive to the timing of poly I:C injection pre-inoculation [85]. Nonetheless, their data suggest that intranasal poly(I·C) should be considered in the development of new treatments for aged individuals at high risk of contracting COVID-19. Second, the migration capacity of DC appears to decline with age [86]. Prostaglandin D_2_ (PGD_2_) expression in the lungs of aged mice correlated with a progressive impairment of respiratory DC (rDC) migration to dLNs, while the blocking of PGD_2_ improves DC migration, T cell responses and survival. In fact, the use of a PGD_2_ receptor antagonist (e.g., ramatroban) has recently been suggested as a means of resolving COVID-19-related immune dysfunction and lymphopenia [87]. Lastly, it should be considered whether disproportionate IL-10 signaling in older individuals could contribute to worse disease outcomes. IL-10 levels were found to increase more drastically in older rats compared to younger rats during SARS-CoV-1 infection [88] and its abundance in patient sera correlates with COVID-19 disease severity [7]. IL-10 is an immunoregulatory cytokine that can induce the differentiation of DC (e.g., moDC) into tolerogenic DC [79], which may lead to impaired T cell activity. IL-10 production may be temporally related to T cell numbers in COVID-19 patients; IL-10, as well as IL-2, IL-4 and TNF-α levels peak and T cell numbers reach their lowest 4–6 days following disease onset [7]. Further investigation of how IL-10 signaling may directly impact COVID-19 outcomes should be conducted, and the age of patients is a factor that should be considered. In general, the production of anti-inflammatory cytokines by DC decreases while their production of pro-inflammatory cytokines increases as individuals age [89]. Therefore, treatments directed toward the blocking of specific inflammatory mediators may be particularly effective in older patients with COVID-19.

A critical initial step of SARS-CoV-2 infection is the virus’ interaction with receptors on host cells that occurs through the S protein on the virus surface [90]. SARS-CoV-2 binds to ACE2 on human cells [91]. However, it appears that additional host factors (such as neurophilin 1, NRP-1) [92] can be involved in the entry step [93,94]. Of interest, our recent study has demonstrated the expression of NRP-1 by immature or mature stimulated DC generated from bone marrow [95]. These findings collectively suggest the S protein–NRP1 interaction on DCs could induce functional responses and be a potential antiviral target to treat SARS-CoV-2 infection.

LL-37 is an antibacterial peptide that could ironically be used in the treatment of COVID-19. It induces DC maturation and the release of type I IFN by APCs [96]. If future work reveals dysfunctional DC maturation in in vivo COVID-19 models, LL-37 may have therapeutic properties to be harnessed. Finally, an aspect of DC function that may be dysfunctional at the metabolic level during COVID-19 is the kynurenine pathway. This pathway of tryptophan synthesis contains all the enzymes necessary for generating NAD^+^ and has received increasing attention for its connection to inflammation. Activation of the kynurenine pathway appears to give rise to immunosuppressive DC and CD8 T cell activity [97], so metabolomic studies of these cell types may characterize a new therapeutic target for COVID-19. In theory, inhibitors of the kynurenine pathway, such as nicotinylalanine and *meta*-nitrobenzoylalanine [98], could help reverse potential immunosuppressive mechanisms at play in COVID-19.

Chloroquine/hydroxychloroquine was first proposed in 2003 for the treatment of severe acute respiratory syndrome (SARS) and its effectiveness as an antiviral drug against SARS-CoV-2 has also been the subject of debate [99]. A simulation performed using serum samples from COVID-19 patients predicts that early administration of hydroxychloroquine increases antigen presentation by DC and enhances the priming of T cells [100].

## 6. Concluding Remarks

To date, most relevant studies of DC activity have been conducted with SARS-CoV-1, although substantial findings are beginning to emerge. Moving towards a thorough understanding of DC functions during COVID-19 may depend on in vivo studies which observe the dynamic interactions between SARS-CoV-2 and the DC subsets discussed in this review, similar to the work of Liu et al. for SARS-CoV-1 [43]. In addition, much can be learned from in vitro infections of these DC subsets with SARS-CoV-2 that measure TLR, IFN and pro-inflammatory cytokine signaling as well as their resultant capacities for migration, maturation, naïve T cell stimulation and trans-infection. Ultimately, the plethora of ways that DC intersect with COVID-19 immunopathology might present us with new and DC-specific targets for drug design and repurposing.

## Figures and Tables

**Table 1:** Diverse roles of DC functions in different DC Subsets.

DC Subset	Potential Roles in Viral Infections
**cDC1**	- IL-12p70 production for the activation of Th1 cells [10] - Strong producers of type I and III IFNs and TNF-α [10] - Potent inducers of CD8+ T cell responses [10]
**cDC2**	- Inducing Th1, Th2 and Th17 cells [10] - Efficient promoters of CD8+ and follicular T helper cell responses [10] - Have various immunoregulatory roles, including the ability to stimulate intestinal Treg cells [11]
**pDC**	- A significant producer of type I IFN [12] - Recruiting NK for antiviral cytotoxicity [13] - Enhancing differentiation and maturation of DC through type I IFN signaling [14] - Producing chemokines to recruit CD4+ and CD8+ T cells to the site of infection [14] - Trigger B cells to differentiate into immunoglobulin-secreting plasma cells [14] - Act as inducers of CD4+ and CD8+ T cells, but less effectively compared to cDC [14]
**LC**	- Being situated in epithelia, LC are one of the first cell types encountered by invading pathogens [15] - Type IIR cytokine-mediated prevention of Ectromelia virus dissemination [15] - Trans-infection of CD4+ T cell by HIV in dLN [15] - Can stimulate B and T cells in response to viral infection [16]
**moDC**	- The bona fide in vivo equivalent of moDC is currently debated [11] - Producers of inflammatory cytokines, including IL-6, TNF-α and IL-1β [17] - Strong activators of CD4+ and CD8+ T cells and Th17 [17]

**Table 2:** Current and potential drugs affecting DCs in SARS-CoV-2 infection.

Treatment	Availability	Mechanisms of Action	Potential Target	Evidences
Anti-S protein	Available	Prevents viral entry, neutralizes virus	DC & Other cells	Prevents trans-infection of SARS-CoV-1 by DC
Anti-IL-6	Available	IL-6 inhibitor, blocks cytokine storm	DC & Other cells	Increases the capacity of DC to activate T-cell responses against SARS-CoV-1
Tocilizumab/Sarilumab	Available	IL-6 receptor inhibitor, blocks cytokine storm	DC & Other cells	Expected to have same effect as anti-IL-6 on DC
Corticosteroids	Available	lower the levels of pDC, cDC, CD4 and CD8+ T cells	DC & Other cells	Limit DC levels, may further delay pDC response
Chloroquine/hydroxychloroquine	Available	Not known, change the pH of endosomes, prevent viral entry, transport, and post-entry events	DC & Other cells	Increases antigen presentation by DC
Mavrilimumab (anti-GMCSFRα)	Available	Decrease leukocyte activation, ameliorate immunosuppression	DC, macrophages, NK, and T cells	GM-CSF promotes tolerogenic DC. Downstream effects resemble multi-system inflammatory syndrome
Type I IFN	Available	Compensate for insufficient type I IFN production by DC	Cells that are permissive to SARS-CoV-2 infection.	Production by pDC is suppressed, is lowest in severest cases.
TLR3 Agonist (poly I:C)	Potential	Stimulate type I IFN production by DC	DC & Other cells	Prophylactic intranasal use is 100% protective for SARS-CoV-1-infected mice
Anti-NRP	Potential	Inhibit viral entry	DC, respiratory and olfactory epithelia	Five out of 6 autopsy samples are positive for both S protein and NRP1
LL-37	Potential	Induces inflammasome activation, IL-1β and IL-18	DC & Other cells	Induces DC maturation and release of type I IFN by APCs
Anti- Kynurenine pathway (nicotinylalanine/meta-nitrobenzoylalanine)	Potential	Strengthen adaptive immunity	DC and CD8 T cells	Pathway drives immunosuppressive activity of DC and CD8 T cell

## Data Availability

N/A.
